# Aspects of triage for infants: a narrative review

**DOI:** 10.1007/s00431-025-06127-3

**Published:** 2025-04-12

**Authors:** Anouk Hopman, Elise S. Talsma, Dennis O. Mook-Kanamori, Arjan B. Te Pas, Ilona C. Narayen

**Affiliations:** 1 Division of Neonatology, Department of Pediatrics, Leiden University Medical Center, Leiden, The Netherlands; 2 Department of Pediatrics, Radboud University Medical Center, Nijmegen, The Netherlands; 3 Department of Public Health and Primary Care, Leiden University Medical Center, Leiden, The Netherlands; 4 Department of Pediatrics, Rode Kruis Ziekenhuis, Beverwijk, The Netherlands

**Keywords:** Triage, Infants, Primary care, Vital signs

## Abstract

Infants under 1 year old frequently visit the general practitioner with acute illnesses. Assessing the severity of illness in this group can be challenging as signs and symptoms may be observed in both sick and healthy infants. Current triage systems are primarily designed for older children and adults and have been validated mainly in high-prevalence settings, such as emergency departments. As a result, these systems often result in undertriage, which can lead to delayed treatment and adverse outcomes.

*Conclusion*: This review reports the existing triage and scoring systems, currently used in infants. We discuss the strengths and limitations of this systems. Furthermore, we explore how the integration of clinical features with vital signs, such as heart rate and oxygen saturation, can improve the accuracy of triage for infants. The BabyCheck, validated for use in primary care for infants under 6 months of age, and the use of pulse oximetry offer promising improvements. Further research is essential to develop and validate an optimal triage system for infants under one year of age in the general practitioner setting.

**Table:** 

**What is Known:** • *Current triage systems are widely used in emergency departments but show limitations, especially when applied to infants.* • *These existing triage systems often result in undertriage or overtriage, which can lead to either unnecessary healthcare utilization or delayed treatment for serious conditions.*	
**What is New:** *• Combining vital signs such as heart rate and oxygen saturation with clinical features, may improve the accuracy of triage systems for infants.*

**What is Known:**

• *Current triage systems are widely used in emergency departments but show limitations, especially when applied to infants.*

• *These existing triage systems often result in undertriage or overtriage, which can lead to either unnecessary healthcare utilization or delayed treatment for serious conditions.*

**What is New:**

*• Combining vital signs such as heart rate and oxygen saturation with clinical features, may improve the accuracy of triage systems for infants.*

## Introduction

Infants up to 1 year of age constitute a higher proportion of visits to general practitioners (GPs) compared to any other age group, with an average of 4.2 visits in the first 6 months of life [1–3]. In developed countries, each child visits the GP more than once a year due to symptoms of acute infections. However, among these children, the likelihood of serious infections is rare, being approximately 1% [4]. This highlights the role of GPs as gatekeepers in health care.

The high utilization of medical services by this group of children can be attributed to the high prevalence of illnesses and the challenge of assessing the severity of illness, as signs and symptoms may be observed in both sick and healthy infants [5, 6]. Visual cues are often used to assess illness severity, and this frequently leads to increased visits to the out-of-hours general practitioner post-following initial triage by telephone. Early identification and treatment are crucial, as the condition of infants can deteriorate rapidly [7].

In addition to the primary care, the emergency department (ED) also experiences a significant number of consultations; in the USA, children account for nearly 25% of all ED visits [8]. Several studies have indicated a substantial portion of non-urgent pediatric ED visits, suggesting the need for a re-evaluation of current referral practices [9, 10].

Both unwarranted referral and delayed referral have detrimental effects. The evaluation process at the ED consumes considerable time for parents, patients, and healthcare providers. Additionally, the pediatric ED is relatively cost-inefficient for cases of low acuity [11]. On the other hand, delayed referral of infants who are actually severely ill may lead to delay in effective treatment and impede a favorable outcome.

Despite being a longstanding issue, there is currently no evidence-based approach for general practitioners to triage and accurately identify or rule out illnesses in children under 1 year of age. This is important, as the GP plays the key role in determining whether a child needs to be evaluated at the ED. The GP’s decision is critical to ensuring timely and appropriate care for children when needed, while preventing unnecessary referrals to the ED.

This review provides an overview of various aspects of existing triage and scoring systems for infants up to 1 year of age. This information can serve as a basis for optimizing the triage system for the general practitioner to assess the severity of illness in infants and determine whether referral to the ED is necessary for further evaluation and care.

## Methods

The information used to write this narrative review was collected from PubMed, Web of Science, and Cochrane. We used search terms related to four components: infant, emergency department, primary care, triage, and level of illness, published between 1990 and 2024.

Studies were included if they focused on triage systems for children or described scoring methods aimed at evaluating the severity of illness in pediatric patients, with a particular focus on infants under one year of age and the use in primary care. The selection process involved an initial screening of titles and abstracts to assess relevance to the topic. Full-text articles were subsequently reviewed to confirm eligibility based on predefined inclusion criteria.

### Existing triage systems

The following triage and scoring systems are commonly used for pediatric patients (Table 1).

**Table 1:** Comparison of the different triage systems for infants

Triage system	Advantages	Disadvantages	Applicability for infants < 1 year
NTS^12^	• Investigated in the primary care setting • Strong association between high urgency levels and hospital admissions/resources uses	• Undertriage in 17.4% of the cases • Overtriage in 20.2% of the cases	• No data about the applicability for infants below the age of one
MTS^14^	• Widely used triage system • Investigated in the primary care setting • Uses combinations of complaints and vital signs	• Moderate validity • Significant rates of undertriage and overtriage • Less accurate for children with chronic illnesses and infants under three months old	• High risk of undertriage for children below the age of 3 months old compared to children aged 8–16 years (odds ratio 9.6) and children aged 3–11 months (odds ratio 2.6) • Less sensitive for very young patients
NICE guidelines^20^	• High sensitivity (85.8%) for the detection of SBI • Investigated in primary care settings	• Low specificity (28.5%) leading to potential overtreatment • Not suitable for primary care due to overtriage risks • Not accurate in detecting serious illness or determining if a child could be managed at home	• In the study by Sukayna et al., 25% of the children were under 1 years of age
• BabyCheck^24^	• High sensitivity and specificity • Investigated in the hospital as well as in primary care Positive feedback from GPs	• Distribution of the BabyCheck to a group of mothers did not affect the use of primary care	• Suitable for infants up to 6 months
PaedCTAS^27^	• Strong association with ICU admissions, length of stay at the ED, hospitalization and resource utilization costs	• Less accurate in predicting certain interventions and admissions compared to previous tools	• Median age in the studies was higher than 1 year • Performance may vary based on the age of the patient
ESI^30^	• Strong validity for prediction hospital admission, ED length of stay, and resource utilization • High interrater reliability	• Variability in triage decision for the most critical and least severe cases as well as children presenting with non-traumatic medial issues	• Variation in triage decisions for patients below the age of 1
PRISA^33^	• Good predictor of the risk for hospitalization in an ED	• More accurate for sicker patients	• Median age was 5.5 years old

#### The Netherlands triage system (NTS)

The NTS is a five-level, computer-based protocol used across various emergency care settings in the Netherlands, including general practitioner cooperatives (GPCs), ambulance dispatch centers, and EDs. Based on expert consensus, the NTS includes 56 presenting problems and 238 triage criteria. A study validating the NTS for pediatric patients in EDs and GPCs showed that higher urgency levels (U1, U2) correlated with increased hospital admissions and medical resource use, while lower levels (U4, U5) often resulted in follow-up care from GPs. Children assigned higher urgency levels were more likely to be referred to the ED [12].

A cross-sectional study assessed the reliability and validity of the NTS for pediatric triage in various emergency care settings, including GP out-of-hours services. In this study, 116 triagist assessed 40 fictional pediatric cases with urgency levels defined by an expert panel. The study found an inter-rater reliability of 0.73, with 62.3% agreement with the reference standard. Undertriage occurred in 17.4%, and overtriage in 20.2%. Sensitivity for high-urgency cases (U0-U2) was 85%, and specificity for low-urgency cases (U3–U5) was 90% [13].

#### Manchester triage system (MTS)

The MTS is a widely used triage system that can be applied to both adults and children. It utilizes a combination of presenting complaints and vital signs to assign patients to different levels of urgency.

There is one study that investigated the use of the MTS in primary care, Van Veen et al. investigated the ability of the MTS to identify less urgent patients in primary care. Additionally, they evaluated the ability of GP services to meet patient needs compared to the ED. They found that the MTS can identify less urgent patients safely, except for children under 1 year of age, patients with dyspnea, gastro-intestinal problems, or fever without identified source [14].

However, many studies evaluated the use of the MTS in the ED. Roukema et al. assessed the MTS urgency levels compared to resource utilization, hospitalization, and a predefined reference classification for true urgency, based on vital signs, resource utilization, and follow-up. They included 1065 patients with a mean age of 4.6 years. They showed that the MTS was neither very sensitive nor very specific in a pediatric population. It had a sensitivity of 63% and a specificity of 78% in detecting emergent/very urgent cases. Undertriage occurred in 15% of patients and overtriage in 40% of the patients [15].

Another prospective observational study by van Veen et al. was performed to validate the use of the MTS in pediatric emergency care. They included 17,600 children and measured the validity by comparing the urgency categories of the MTS with a predefined independent classification of urgency. Nurses applied the MTS in 95% of the children who visited the ED, with an overrule of the urgency category in 10% of cases. The MTS agreed with the reference standard in 34% of the children, with overtriage occurring in 54% and undertriage in 12%. They concluded that the MTS had moderate validity but erred on the safe side, leading to more overtriage than undertriage when compared to an independent reference standard for urgency. Additionally, the study found that the MTS was less sensitive for very young patients (0–2 months), with a sensitivity of 50%, while specificity was better for older children (> 4 years) [16].

Zacharisse et al. examined the safety of utilizing the MTS in pediatric emergency care for identifying children in need of intensive care unit (ICU) admission. They found that 28.7% of the infants admitted to the ICU were undertriaged by the MTS. They identified serval risk factors associated with undertriage for example: age below 3 months, medical presenting problem, comorbidity, referral by a medical specialist, and presentation during the evening or night shift [17].

The risk of undertriage in the younger age group was shown in different studies. One study found that in total, when using the MTS at the ED, 0.9% (119) of infants were undertriaged. These cases were discussed by experts and they found that 50% of these cases might experience at least one consequence because of undertriage. Examples of consequences are treatment delay, longer hospitalisation, complications, morbidity, and mortality. They found that infants below the age of 3 months have the highest risk of undertriage, with an odds ratio of 9.6 compared to children aged 8–16 years. This odds ratio was 2.6 for children aged 3–11 months [18].

#### NICE guidelines

In 2007, the NICE developed the traffic light system to identify children who are at risk for a serious infection and they updated the guideline in 2019. This system categorizes symptoms into low (green), moderate (amber), or high (red) risk levels for serious illness, guiding clinicians in assessment. The guidelines recommend that “red” category children be referred to pediatric specialists, while “amber” cases require clinical judgment for hospital referral [19].

A recent study investigated the traffic light system in primary care, including 6797 acutely ill children under five. It found 32% had red flag symptoms, 62% amber, and only 6% were green or uncategorized. However, of the children with at least one red symptom or sign, only 1.6% was referred for assessment on the same day. All children classified as red should be referred for some day assessment if the NICE guidelines were followed. They concluded that the traffic light system is not suitable for primary care because it will overtriage the need for assessment at the ED [20].

Clark et al. assessed the traffic light system’s accuracy in 6703 children under five. They found 31.6% categorized as red, 62.7% amber, and 5.7% green. Of those, 2.1% were hospitalized within 7 days, with 12.2% (0.3% overall) having a serious illness. The system showed 58.5% sensitivity and 68.5% specificity, leading to the conclusion that it is inaccurate for detecting serious illness in primary care and should be updated or replaced [21].

The study from Sukanya et al. assessed the effectiveness of the traffic light system in accurately identifying three prevalent serious bacterial infections (SBI) in young febrile children visiting the pediatric ED. A total of 15,781 children under 5 years of age presenting with febrile illness were included. A quarter of these children were under the age of 1 year old. The traffic light system showed 85.8% sensitivity but only 28.5% specificity for SBIs. Adding urine analysis improved accuracy. They concluded that while the system has moderate sensitivity, its low specificity necessitates careful clinical judgment to avoid overtreatment and unnecessary investigations [22].

#### The babycheck

In Great Britain, the BabyCheck was developed as a clinical tool to assess illness severity in infants under 6 months. It includes 19 questions that quantify illness severity, with higher scores indicating sicker babies [23]. Morley et al. found 92% of GPs considered BabyCheck accurate, and 74% found it helpful, though some found it less useful in non-ill cases [24]. Thornton et al. studied its use by junior pediatricians and found it matched the registrar’s assessments with 87% specificity [25]. BabyCheck was also seen as practical by parents and GPs. However, a randomized trial found that distributing BabyCheck booklets to mothers did not reduce GP visits, though a more thorough implementation might enhance its effectiveness.

#### The pediatric Canadian triage and acuity scale (PaedCTAS)

The PaedCTAS triage system evaluates physiological factors (appearance, neurological status, respiratory rate, heart rate, and perfusion) and symptom combinations to determine triage levels, ranging from level I (resuscitation) to level V (non-urgent).

A before-and-after prospective study assessed the performance of the PaedCTAS compared to previously used triage tools. Their previous tool was a 4-level triage system developed by pediatric healthcare experts. This tool categorizes patient into four levels of severity: emergent, urgent, less urgent, and non-urgent. The study found that PaedCTAS triaged more patients into higher acuity levels (53% vs. 36%, *P* < 0.05) but was less accurate in predicting admissions and certain interventions. There were no significant differences in the prediction of other outcomes, such as blood culture and intravenous fluid bolus. The mean Pediatric Risk of Admission (PRISA) scores were similar between the two groups. They concluded that the PaedCTAS did not perform better than their previous triage tool [26].

A retrospective cohort study involving 550,940 infants with a median age of 47 months from 12 EDs found a strong association between triage level and ICU admission; 79% of the patients admitted to the ICU were triaged at level 1 or 2. The PaedCTAS triage level was also associated with the length of stay at the ED and hospitalization. However, patients triaged at level 1 had the same length of stay at the ED compared to level 2. They concluded that there was a strong association between triage level and multiple markers of severity. The researchers recommended further studies to identify scenarios where PaedCTAS may perform suboptimal, possibly due to factors like patient age, chief complaints, or setting [27].

Another study assessed the relationship between resource utilizations costs in the ED and the PaedCTAS. The median age of the infants was 64 months. The results showed that the PaedCTAS correlates well with laboratory investigation costs, where acute PaedCTAS categories exhibited higher costs. Additionally, there is a strong correlation between higher acuity levels and increased imaging costs [28].

#### The emergency severity index (ESI)

The ESI is a 5-level triage system developed by a team of ED physicians and nurses to prioritize patients based on acuity and predicted resource utilization. The latest version is called ESI version 4 (ESI v.4), which considers both patient acuity and expected resource needs. In ESI v.4, patients are categorized into five levels. Levels 1 and 2 represent the highest acuity and priority cases, while levels 3, 4, and 5 are based on expected resource requirements. ESI v.4 is the most updated version and has been expanded to include fever as a criteria for pediatric patients [29].

Travers et al. conducted a study evaluating the reliability and validity of the ESI for pediatric triage at five different sites. Their research aimed to determine the accuracy and effectiveness of the ESI in assessing the severity of pediatric patients’ conditions in ED settings. They demonstrated an interrater reliability of 0.77. Variations in triage decisions were observed for patients with the most critical conditions as well as those with less severe conditions. Inconsistencies were also noted for patients below the age of 1 compared with patients from age 1 to 17 years and those presenting with medical issues rather than traumatic complaints [30].

A study assessed the validity and reliability of ESI v4 in predicting hospital admission, ED length of stay, and resource use among 780 patients (median age 5.4 years). Patients were divided into higher-acuity (ESI levels 1–3) and lower-acuity groups (ESI levels 4–5). The higher acuity group had a significantly higher admission rate (21% vs. 0.96%), longer ED stays, and greater resource utilization. The interclass correlation coefficient was 0.96 between pediatric triage nurses and 0.91 between nurses and physicians. The authors concluded that ESI v4 is a valid predictor for pediatric ED outcomes [31].

#### The pediatric risk of admission score (PRISA)

The PRISA was described by Chamberlain et al*.* It is a score which can predict the risk of children being hospitalized based on their initial evaluation. The score is based on 21 components gathered from medical history, physiological data, and therapies. The PRISA score has a range from 0 to 97. All the components of the PRISA are objective and can be easily retrieved from the patients’ charts [32].

A prospective study was performed to evaluate the value of the PRISA score. They included 1930 patients from a pediatric tertiary center ED. Among these, 203 hospitalizations were observed. The PRISA score predicted 235 hospitalizations. Two deaths occurred within the study population. Both patients were classified in the highest decile of the probability of admission. They found the PRISA score to be a good predictor of the risk for hospitalization in an ED. However, it seemed to be more accurate for the sicker patients [33].

Another study was performed by Chamberlain et al. to validate the PRISA score in a multi-institutional recalibration. They enrolled 3273 patients with a mean age of 5.5 years. In the validation sample, 122.1 admissions were predicted and 110 observed. Additional studies will be needed to determine the extent to which PRISA is directly applicable to other emergency department settings (e.g., non-tertiary, small-volume hospitals) [34].

#### Combination of clinical features instead of single alarm symptoms

The estimation of illness severity in infants is influenced by various factors. Early and accurate diagnosis of serious illness is essential. In order to detect possible serious illness, physicians often look for the presence of so-called “red flags.” However, red flags occur infrequently, even in children with a serious infection, and no single clinical feature can rule out serious infection. The combination of symptoms should be used to exclude the possibility of a serious infection [35].

Most studies examining the effectiveness of alarming signs in identifying serious infections were conducted in secondary care setting. Furthermore, the alarming signs that are used by GPs to assess a child are also based on research performed in secondary care only. A study investigated alarming signs in febrile children in primary care found that 50% of all children had one or more alarming sign. However, we it is known that the incidence of serious infection is low in primary care. Therefore, the frequent occurrence of alarming signs would likely result in a high rate of false-positive prediction for infections. The authors suggest that alarming signs in primary care should be related to the prognosis of the disease instead of the presence of a serious infection [36].

By incorporating a combination of clinical features and looking into the severity of disease instead of only assessing the presence of a serious infection, triage systems can provide a more comprehensive assessment of infants’ health status. This could potentially improve the accuracy of identifying children in need of further medical care.

#### Use of vital parameters

Heart rate (HR) and oxygen saturation (SpO_2_) are objective parameters, with low SpO_2_ serving as early indicator of potential infections, respiratory issues, or circulatory pathology in infants [37]. Infants under 2 months of age often present with clinical features of serious illness that are subtle and nonspecific, potentially leading to undertriage [38].

Studies demonstrated that including vital signs in assessments could reduce undertriage. One study found that systematic assessment of vital signs in infants under 3 months could prevent 21% of clinically severe undertriage [18]. Another prospective cohort study of 700 children found that those with serious or intermediate infections were more likely to have fever, tachycardia, SpO2 below 94%, and prolonged capillary refill. Authors concluded that abnormal vital signs offer diagnostic performance comparable to triage systems like the MTS and NICE traffic light system for identifying serious infections [39].

Zachariasse et al. proposed updating the MTS with vital signs, showing a 0.7% decrease in undertriage (200 patients) when age-specific adjustments were incorporated. They concluded that using vital signs like HR and respiratory rate enhances MTS effectiveness [40].

However, there are also studies that showed that the use of vital signs can lead to overtriage. A study found that 13.6% of pediatric ED visits were overtriage due to tachycardia. This effect was most pronounced in children aged 3 months to 9 years. HR in young children can be easily influenced by the stressful ED environment [41].

While HR and SpO2 have additive value in illness detection, evaluating oxygen levels by skin color can be challenging, and auscultating HR is often inaccurate. Pulse oximetry (PO) is recommended for quantifying these parameters. Studies show that PO is feasible in infants and acceptable to parents. Considering the added value of these objective parameters and the safety and feasibility of PO, it can be a valuable tool when assessing infants [38, 42, 43].

## Conclusion

Current triage and scoring systems used in infants are primarily designed and validated for older children and adults, and has been shown to be less effective for infants. This can lead to challenges in assessing illness severity and may result in undertriage, potentially causing treatment delays and adverse outcomes. Moreover, age-specific considerations are important, as infants have distinct clinical presentations. Additionally, there is limited research in primary care settings, despite infants constituting a significant portion of GP visits.

To address these challenges, there is a need for a triage system that combines clinicals features with vital signs like HR and SpO_2_ levels. The BabyCheck would potentially be a suitable triage tool, as it has been validated for infants and in primary care settings. Moreover, measuring HR and SpO_2_ via PO seems to offer potential for improving accuracy of infant triage. However, it is important to further investigate whether it is feasible for triagists in primary healthcare settings to reliably measure these vital signs in infants. Triagists require experience to obtain accurate measurements, and the appropriate equipment with the correct probes must be available. Further research is needed to assess this.

## Data Availability

No datasets were generated or analysed during the current study.

## Abbreviations

ED: Emergency department
ESI: Emergency severity index
GP(s): General practitioner(s)
GPC(s): General practitioner cooperatives
HR: Heart rate
MTS: Manchester Triage System
NICE: National Institute for Health and Care Excellence
NTS: The Netherlands Triage System
PaedCTAS: The Pediatric Canadian Triage and Acuity Scale
PO: Pulse oximetry
PRISA: Pediatric Risk of Admission Score
SBI: Serious bacterial infection
SpO2: Oxygen saturation
